# Fractal Dimension as a Tool for Assessment of Dental Implant Stability—A Scoping Review

**DOI:** 10.3390/jcm11144051

**Published:** 2022-07-13

**Authors:** Sukanya Mishra, Manoj Kumar, Lora Mishra, Rinkee Mohanty, Rashmita Nayak, Abhaya Chandra Das, Sambhab Mishra, Saurav Panda, Barbara Lapinska

**Affiliations:** 1 Department of Periodontics and Oral Implantology, Institute of Dental Sciences and SUM Hospital, Siksha ‘O’ Anusandhan University, Bhubaneswar 751003, Odisha, India; drmishrasukanya@gmail.com (S.M.); rinkeemohanty@soa.ac.in (R.M.); rashmitanayak@soa.ac.in (R.N.); drabhaya2011@gmail.com (A.C.D.); sauravpanda@soa.ac.in (S.P.); 2 Department of Conservative Dentistry and Endodontics, Institute of Dental Sciences and SUM Hospital, Siksha ‘O’ Anusandhan University, Bhubaneswar 751003, Odisha, India; loramishra@soa.ac.in; 3 Department of General Surgery, SCB Medical College and Hospital, Mangalabagh, Cuttack 753007, Odisha, India; sambhab@gmail.com; 4 Department of General Dentistry, Medical University of Lodz, 92-213 Lodz, Poland

**Keywords:** fractals, fractal analysis, dental implants, osseointegration, implant stability

## Abstract

A lot of modalities for assessing implant stability are available for clinicians, but they fail to assess trabecular changes as they are solely dependent on the operator’s skills. The use of Fractal Dimension (FD) has evolved to be used as a measure for trabecular changes depicting implant stability before and after implant placement. The objective of this systematic review was to qualitatively analyse the available scientific literature describing the use of FD as a tool to measure implant stability on the basis of trabecular changes. An electronic search in PubMed, Web of Science and Scopus was carried out using relevant keywords, such as: fractal dimension; fractal analysis; dental implants; implant stability; osseointegration, etc. Studies reporting the use of FD as a tool to measure implant stability were included and subjected to qualitative analysis using ROBINS-I and Cochrane risk of bias assessment criteria. Fourteen studies were included in this review. Results showed that FD was found to be used solely as a measure of implant stability in seven studies, out of which six studies showed an increment in FD values. The majority of studies concluded with a statistical correlation between FD and respective other assessment methods used. FD may not serve as a sole indicator of implant stability; however, it can be used as an adjunct to conventional methods along with additional fractal factors.

## 1. Introduction

Radiographic evaluation has been a crucial requisite in the diagnosis of peri-implant tissues for gauging the long-term success or failure of dental implants [1]. It can be interpreted by identifying the peri-implant marginal bone loss and radiolucency. The radiographic procedure is widely used because of its non-invasiveness and convenience of being performed at any stage of the procedure, apart from the limitations of being two dimensional and of low image resolution [2].

Implant stability is characterized by a lack of clinical mobility, the presence of which can cause fibrous encapsulation and failure of the dental implant [3]. A well seated implant with adequate stability is the pre requisite for accomplishment of proper osseointegration and its long life [4]. Widely used mechanical methods of implant stability are resonance frequency analysis (RFA) [5], Periotest [6], insertion torque [5] and percussion test [7]. However, these methods do not give a proper idea of the changes occurring at the trabecular level during osseointegration [8]. Advanced techniques have therefore been introduced, e.g., cone beam computed tomography (CBCT), micro-CT, fractal analysis, Feret diameter (FeD) analysis and Finite element method [9].

Fractal dimension (FD) is the representation of a self-similar geometrical structure when the object/image is processed after fractioning it into various sections [10]. The dimensionality of FD measurement depends of the object measured. Similarly to whether a point, or a line, or a 2-dimensional plane or a 3-dimensional model is measured, FD values will be changed in accordance, respectively. It has been utilized in assessing the trabecular structure of mandibular condyles of patients with temporomandibular disorders, for characterization of sialographic images, mandibular bony healing after orthognathic surgery, etc. It can therefore be used as a method to detect the trabecular changes during and after implant placement. It is therefore an indirect and non-invasive form of bone density measurement. A systematic review was conducted by Kato et al. [11] to assess the use of fractal analysis (FA) in dental images. Most of the articles included in that review showed significant differences in the comparison of healthy and sick patients.

There is an unclear relationship between the quality of bone and primary implant stability. Of all the methods used until now, RFA has been used widely for implant stability measurement. However, there is weak evidence to support the relationship between implant stability and bone density. Until now, systematic reviews on the use of RFA and insertion torque are conducted, which aided only in assessing whether there is any mobility present in the implant that was inserted [12,13]. Therefore, there is a need for the prediction of implant stability at planned implant sites, based on the quantitative measurement of bone density, as well as a need to quantify the changes simultaneous to bone remodeling. So, as an alternative method, fractal analysis can be used to evaluate trabecular changes quantitatively.

The objective of this systematic review is to provide scientific evidence to determine whether Fractal Dimension can be used to measure implant stability by quantitative measurements of bone density at planned implant sites.

## 2. Materials and Methods

This systematic review was carried out in accordance with the Preferred Reporting of Systematic Reviews and Meta-analyses (PRISMA) statement [14]. The protocol for this review was prepared in priority to facilitate the smooth conduction of the review. The systematic review aimed to answer the research question: Can pre-operative, intra-operative and post-operative assessment of FD be used as the sole indicator for dental implant stability?

The following PECO criteria were framed to aid in research question formulation and careful selection of studies: Population (P): Systemically healthy patients undergoing dental implant therapy Exposure (E): Use of Fractal Dimension in the assessment of bony trabeculae for measuring implant stability; Comparison (C): None or any other modality for measuring implant stability; Outcomes (O): Measure of implant stability relative to the change in trabecular bone formation.

### 2.1. Search Strategy

A digitalized search was carried out in PubMed, Web of Science (WoS) and Scopus database to identify the reports to answer the research question. The search strategy was developed using a series of relevant keywords. The electronic search was carried out until April 2022. No limits or restrictions were used to identify the reports. Additionally, a manual search was also carried out in peer-reviewed, international implant-related journals: COIR, CIDRR, JOMI, BDJ, and JPIS. The bibliography of related systematic reviews and potentially eligible articles were also screened for possible reports. The reports retrieved from both electronic and manual searches were then imported into a citation manager (EndNote v7.0, Clarivate Analytics, New York, NY, USA) to discard the duplicates identified within multiple databases. The details of the search along with the keywords used are provided in Table 1.

### 2.2. Study Selection

The reports were then screened based on title and abstract to identify potentially eligible articles and were subsequently subjected to full-text assessment.

#### 2.2.1. Inclusion Criteria

The following inclusion criteria were applied for study selection:

- Clinical studies, Randomized or Non-Randomized controlled clinical trials (RCTs), and both prospective or retrospective studies assessing implant stability with FD and other modalities were included in the review.
- Patients included in the studies should have undergone either immediate or delayed implant placement, either in maxillary or mandibular sites.
- Studies undertaking any adjunctive therapy to implant placement were also taken into consideration.
- Studies employing FD as a measure for implant placement assessed with the use of radiograph taken both at baseline, during, or post-implant placement.

#### 2.2.2. Exclusion Criteria

The studies recruiting patients with any systematic disease, and with poor oral hygiene status, pregnant and lactating mothers, and also having subjects undergoing any chemo- or radiotherapy in the pool were excluded. The studies published in languages other than English and did not report relevant outcomes were also excluded from this review.

**Figure 1:**
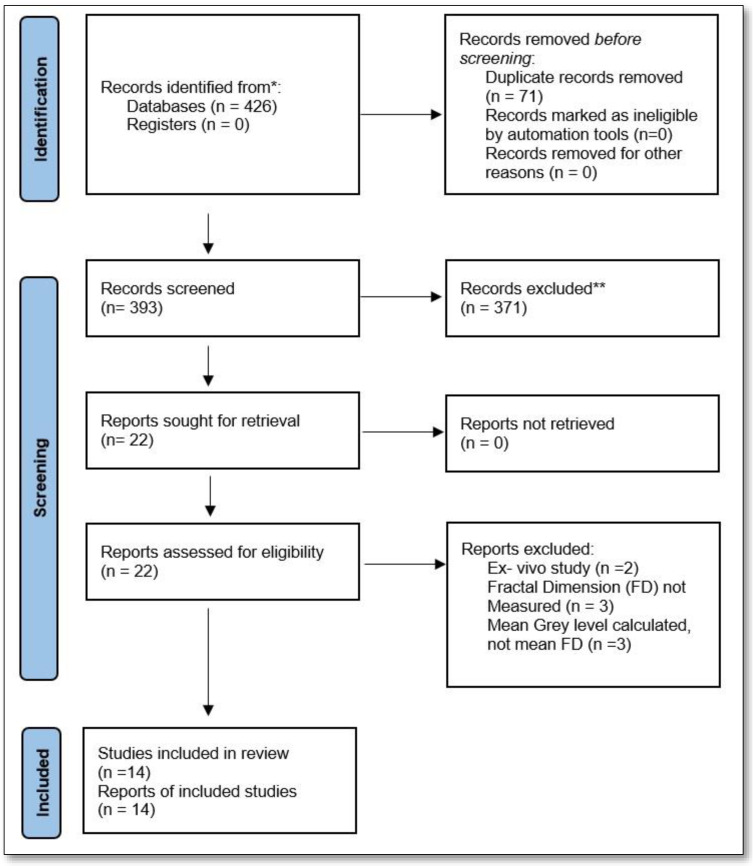
PRISMA flowchart depicting the identification of studies via databases and registers. * Records identified from MEDLINE, PubMed, Scopus and Web of Science databases. ** Records excluded by reviewer only. No automation tools were used.

The study selection was by two independent reviewers (S.M., M.K.) by assessing the title and abstract of each article. The articles meeting the inclusion criteria were selected. In the second screening, full-text articles were selected, taking into consideration the same inclusion criteria, and a final list of articles was selected. In case of any difference in opinion, an open discussion was conducted and in case no consensus was made, the help of a conciliator (S.P.) was taken to come to a decision. The selection and retrieving process is summarised in Figure 1. In total, 426 articles were retrieved and in total 14 articles were scrutinized as final articles.

Data extraction was completed independently by the chief investigator onto an Excel sheet (Microsoft Windows 10) in two categories, demographic and quantitative data, for each study as follows: type of study design, method of randomization, oral hygiene status, presence/absence of smoking habit, the comparison used, sample size, number of implants placed and analysed, type of implant used, time of implant loading, surgical technique used, implant dimensions, healing time, whether any grafts used, type and time of radiograph taken, region of interest (ROI; site and size), method of FD calculation, statistics used and results regarding FD, i.e., FD value and standard deviation of respective ROI.

Since the data extracted were heterogeneous, a qualitative synthesis of every research article was completed.

## 3. Results

Of all the searched articles, which were 426 in total, 14 were found in MEDLINE through PubMed, 395 articles were found in Scopus and 17 articles were found in the Web of Science database (Table 1). Duplicate articles, 71 in number, were removed and the initial screening resulted in 390 articles. However, of those selected, most of the articles used the analysis other than FD, some were review articles and a few others were a letter to the Editor, case reports, conference proceedings, other image analyses, etc., and were excluded. This resulted in 22 full-text articles being downloaded. Of the full-text articles, eight were excluded (Figure 1) and 14 articles were finally taken into consideration for the systematic review.

Out of 14 included articles, four studies were conducted in Turkey [15,16,17,18] two studies were conducted in India [19,20] and others were commenced and ended in South Korea [21,22], Poland [23], Netherlands [24], Italy [25], UAE [26,27] and USA [28]. The articles selected were within the range of the year 2010–2022. Since not many RCTs have been performed in this field, other types of clinical studies were considered. After the selection of the articles, only two RCTs were included in the review [19,27], wherein the method of randomization was mentioned in both the studies. Eight articles were prospective clinical studies [15,22,23,24,25,26,28], while four studies were of retrospective type [16,18,20,21] (Table 2).

Seven studies compared FD analysis with RFA, since RFA is considered to be the most feasible and reliable method for implant stability determination [16,19,22,23,25,26,27]. Only one study considered a different comparison, i.e., a quantitative parameter for calculation of trabecular changes, i.e., lacunarity and Feret diameter [15].

Totalling all the studies, the number of patients evaluated was 393 in number. Out of 393, 374 patients were included in the study as some were lost to follow-up and some didn’t fulfil the inclusion criteria. The total number of implants analysed was 694 and the total number of implants analysed in the comparison was 355 in number.

Out of these, three articles did not mention gender distribution [15,23,28]. In the studies, which have mentioned the gender distribution, the larger sample of the population were males which were 219 in number, whereas female subjects were 161 in number [16,17,18,19,20,21,22,24,25,26,27]. The age range included was within the lowest range of 18 years and the highest age was of 80 years.

When two main factors affecting the outcome of implant stability, i.e., smoking and oral hygiene status were considered, four studies excluded smokers [14,18,21,22] and again five studies excluded subjects with poor oral hygiene [16,19,21,26,27].

In total, 14 studies were included in the review, of which seven studies were prospective clinical trials [15,22,23,24,25,26,28], five were retrospective clinical trials [16,17,18,20,21], and two were RCTs. While considering RCTs, the randomization method was not mentioned in one of the RCTs [27], whereas it was mentioned in the other [19].

The quantitative data extraction as tabulated in Table 3, interprets that the surgical technique used was not mentioned by six articles [15,18,24,25,26,27] and also three studies did not specify what type and brand of the implant were used [15,20,26]. Implant loading time was mentioned in all the studies. It was reviewed if any other intervention was provided along with implant placement and it was found that one study had provided ultrasonic treatment along with implant [27] and two studies incorporated grafts [19,24]. The most important characteristic, i.e., the bony trabeculae was not observed and mentioned by seven articles [16,19,20,21,22,23,25].

The FD was calculated on orthopantomograph (OPG) in six studies [15,16,17,18,20,22], on intraoral periapical radiograph (IOPAR) in six studies [19,21,23,26,27,29], and the other two studies used cone-beam computed tomography (CBCT) [28] and lateral cephalometric radiograph, respectively [24].

The radiographs were taken at 0, 2, 3, 6, 12, 18 and 36 months with maximum radiographs taken at the baseline and three months indicating the time of implant placement and probable time for osseointegration, respectively. However, Veltri et al. [29] and Suer et al. [16] have taken radiographs only after osseointegration, i.e., after 36 months and 18 months, respectively, to check the secondary implant stability.

The ROI for FD calculation was mentioned in every article. Only one article [16] did not mention the dimensions of ROI. Four articles took the whole implant length as the ROI [16,24,26,27], and three studies have selected the mesial and distal aspect of the first macro thread as the ROI [21,27,29]. Two studies have selected the apical portion of the implant as the ROI [20,28]. The rest of the studies have considered the mesial and distal part of the neck, apex, shoulder, and mid-level of the implant, adjacent bone of the implant neck as well as an ROI [15,17,18,19,23].

Seven studies have compared the assessment of implant stability using FD with RFA [16,19,22,23,26,27,29], one study has compared FD with Feret diameter and lacunarity [15], while the rest of the studies have not compared FD with anything, and have assessed implant stability solely using FD [17,18,20,21,24,28].

The software mostly used in the studies was ImageJ (developed by the National Institutes of Health and the Laboratory, U.S.A.) software. One study hasn’t reported the software used for FD analysis [18]. The studies have mostly used the ‘Box- counting Method’ for FD calculation, but two studies haven’t mentioned the method of calculation as tabulated in Table 3 [23,24].

Various outcomes for the relation of FD with the trabecular bone after implant placement were recorded. The clinical trials conducted by Lee et al. [22] and Hayek et al. [26] found a statistically significant correlation between the ISQ values and FD values. However, a low correlation was found in the case of maxillary molars (*p* > 0.01). Kulczyk et al. [23] found the mean FD of ROIs in the mandible to be approximately 1.57. Mean ISQ values were higher in the mandible than in the maxilla. When compared in individual locations linear correlation was found in all ROI, but it was statistically significant only in the neck region. The results of a clinical study by Verhoeven et al. [24] showed no statistically significant differences in any of the parameters close to and away from the implant. FD and RFA values were compared by Abdulhameed et al. [27] using IOPAR in a randomized trial and the results concluded that FD can be used as an adjunctive for the measurement of implant stability. In the clinical trial by Gonzalez-Martın et al. [28] the mean FD change after six months was recorded as 3%, i.e., 1.327 at baseline and 1.368 after six months. Mu et al. [21] conducted a retrospective study, where the mean FD increased significantly to 1.4329 ± 0.0479 from 1.4213 ± 0.0525 after one year, concluding that there was adaptive remodelling. Sansare et al. [20] reported that FD can be used as a prognostic tool for the measurement of implant stability. However, the values showed no statistically significant differences in the measurement of FD, FeD, or lacunarity in the study by Önem et al. [15]. Retrospective studies by Suer et al. [16] and Soylu et al. [18] found a positive statistical correlation between the measurements, but a retrospective trial by Zeytinoğlu et al. [17] found no significant statistical changes. Diana et al. [19] in an RCT evaluated the clinical and radiographic changes concerning bone quality in immediate cases of implants augmented with Platelet Rich Fibrin (PRF). Initially, there was a decrease in bone height, after one month, it increased gradually during the whole course of follow-up. Veltri et al. [25] investigated the correlation between damping and FD of an osseointegrated implant and concluded with no significant correlation.

### Risk of Bias

Since the SR constituted maximum non-randomized clinical trials, the ROBINS-I Risk of Bias Tool was used for risk analysis of the studies. The ROBINS-I tool for NRSI is based on the QUADAS 2 tool to evaluate the diagnostic accuracy of the interventions used and also to evaluate the internal validity of the studies [30]. RoB analysis of the randomized studies was completed using RevMan 5.3 software (Cochrane Group, London, UK). In the study by Abdulhameed et al. [27], the method of randomization was not mentioned. Allocation concealment was not conducted in either of the included studies. While one study was double-blinded, the other one was a single-blinded study. No other bias apart from the before mentioned lines was found (Table 4).

## 4. Discussion

The structured review described the use of FD in dental radiographs to determine the implant stability and it was seen that the maximum number of studies used IOPA radiograph after implant placement followed by OPG. Only one study has used CBCT [28]. In one of the studies, an oblique lateral cephalometric radiograph was used, which has very little application in implantology [24]. The study claimed that it was easy to obtain reproducible changes in a large part of the mandible/maxilla using an oblique lateral cephalometric radiograph and hence it was easy to observe the densitometric changes after implant placement. Calculation of FD using CBCT is recent and novel in the field of implantology and not many studies have been conducted using CBCT, although it is one of the best methods for measurement of trabecular bone width, height, and dimensional changes [31]. Unlike IOPAR, lateral cephalometry, and OPG, it doesn’t absorb the X-ray in the focal region, hence eliminates distortion and overlapping [32]. A simple X-ray provides two-dimensional radiography where the original architecture of the bone is not recorded. A 3-dimensional image of the internal bone structure can be seen in CT scans, MRI scans, and CBCT.

The articles reviewed showed that most of the articles had considered FD in radiographs taken at baseline, suggesting that primary implant stability was mostly evaluated. Three studies considered only secondary stability; the maximum time at which secondary stability was elicited was at 36 months after placement of the implant [25]. The rest of the studies measured both primary as well as secondary implant stability. It is preferable to calculate FD at baseline, i.e., primary stability as well as after a few months for secondary stability, to know the trabecular changes during osseointegration. This goes in accordance with the study by Heo et al. [33] who evaluated the radiographic alterations of the treatment site and found out that the FD value resulted to be low just immediately after surgery and gradually increased with bone remodelling. Although quantitative characteristics can be calculated using FD, its use in initial or primary implant stability prediction is debatable. While the most clinically used is RFA, there is minimum data and scientific evidence on the relationship between bone-implant interface and ISQ values. Also, there is different interpretation leading to uncertainties because of the observer subjectivity. There is a requirement for quantitative methods to keep a check on subjective variation in ISQ interpretation.

The software mostly used for FD analysis was Image J software, available in a public domain as https://imagej.nih.gov/ij/ [34]. It is an image analysis program used extensively in biological sciences. The advantages of the software are its ease of use, extensible plug-in architecture and recordable macro language. A sequence of the process was followed by the authors. High-resolution and compressed images were saved, after extraction of the binary image using the software. The sequence that was followed is cropping of the ROI, then, duplication of the ROI and removal of large-scale variations in brightness with a blurred Gaussian filter (sigma = 35 pixels, kernel size = 33 × 33). Subsequently followed by subtraction of ROI from the original image, then 128 grey values were added to each pixel location, binarization was completed; erosion, dilatation, inversion and skeletonization were also events in this sequence [35].

The placement of ROI was found to be more difficult than selecting ROI size. ROI selected was mostly the whole implant area [22,24], followed by mesial and distal areas adjacent to the first macro thread of the implant screw [21,25,27]. Although no proper study has been conducted to evaluate which trabecular area should be calculated for changes in FD to measure the implant stability. A two year study conducted by Wilding et al. [36] found a significant increase in FD around implants specifically around the neck.

Until now, RFA is supposed to be the most widely used and reliable method of implant stability measurement. Within the seven studies included in the present review that compared FD with the RFA, five studies found a significant statistical correlation between FD and RFA measurements. However, in the study by Abdulhameed et al. [27], the result showed that a significant correlation was present between FD and RFA values only with one ROI, whereas in the other ROI the correlation was non-significant.

Insertion of the implant is not a simple procedure. It is rather accompanied by various pre-insertion steps, such as determination of the site of insertion, presence of any bony defects, measurement of the width of the alveolar ridge, bone density, etc. [37,38]. Therefore, according to the requirements, implant therapy has to be compiled with other treatments for better healing [39]. This might affect the FD values. Also, implant surface treatments [40] might affect the FD measurements. Hence, these factors can be taken into consideration while FD analysis.

One of the factors which determines the stress distribution around the implants is whether it’s a tissue level or a bone level implant. Various studies have shown that bone level implants have higher stress distribution around them as compared with tissue level implants [41,42] reducing early failure risks. The prosthetic design also has a bearing on implant stability. Based on the scarce data we have, it could be said that properly adjusted splinted and milled prosthesis should be used to prevent excessive loading [43].

Despite all the advantages, FD analysis comes with its share of demerits. One of the primary limitations is the dimension of ROI. Whatever dimension the ROI is chosen, it is difficult to visualise the complete implant length on IOPARs. RFA represents the whole length of the bone-implant interface however; the chosen dimension of ROI doesn’t represent it. This was contradicted in studies by Meredith et al. [44] and Pattijn et al. [45]. They demonstrated that when a proper fixation of an implant is present, an increase of implant length embedded in the bone does not affect RFA significantly. In other words, given a stiff interface, it seems that resonance frequency is not affected by the implant length inside the bone. As a result of this, it could be supposed that vibration response can be correlated with a shorter ROI. Another drawback comes with the means to obtain radiographic images, i.e., conventional or digital, the conventional one is later converted to digital. So, both might differ in regards to the image resolution, giving different values of FD. The third limitation is the method of calculation of FD. Different calculation methods can yield different FD values. The most commonly used are the box-counting method and pixel-dilation method. However, there is no reliable source to confirm the best method of FD calculation, although, in this review, all the articles have used the box-counting method. It was also observed that some studies suggest an increase in FD with a decrease in bone density while other studies suggest vice versa [16]. This might be due to the complex nature of the bony trabeculae.

Other modalities of radiographic analysis can also be used along with FD for more accurate measurements such as the Finite element analysis and FeD analysis [20]. The Finite element analysis that measured the trabecular changes with strain was also compared and was found analogous with FD [46].

## 5. Conclusions

With the ever changing field of research, the utilization of the FD has significantly increased in the field of dentistry. The included studies concluded that FD analysis can be used as a viable and easily accessible method for bone quantification, however, due to its novelty, until now there is no consensus on a standardized protocol for FD calculation. Therefore, FD cannot be used as a single methodology of measurement of implant stability. It can be either used as an adjunct to the widely applied RFA or additional fractal factors such as Feret diameter or lacunarity.

## Figures and Tables

**Table 1:** Search Strategy for each electronic database.

Database	Search String	No. of Articles Retrieved
PubMed	(((“fractal dimension”[All Fields]) OR (“fractal analysis”[All Fields])) AND (((((“dental implants”[All Fields]) OR (“implant stability”[All Fields])) OR (“osseointegration”[All Fields])) OR (“bic”[All Fields])) OR (“bone to implant contact”[All Fields]))) AND ((((((((((“periapical radiograph”[All Fields]) OR (“iopa”[All Fields])) OR (“panoramic radiographs”[All Fields])) OR (“opg”[All Fields])) OR (“orthopantomograph”[All Fields])) OR (“cbct”[All Fields])) OR (“cone beam computed tomography”[All Fields])) OR (“subtraction radiography”[All Fields])) OR (“computed tomography”[All Fields])) OR (“ct”[All Fields]))	14
Scopus	((ALL (fractal AND dimension) OR ALL (fractal AND analysis))) AND ((ALL (dental AND implants OR ALL (implant AND stability) OR ALL (osseointegration) OR ALL (bic) OR ALL (bone AND to AND implant AND contact)) AND ((ALL(periapical AND radiograph) OR ALL (iopa) OR ALL (panoramic AND radiographs) OR ALL (opg) OR ALL (orthopantomograph) OR ALL (cbct) OR ALL (cone AND beam AND computed AND tomography) OR ALL (subtraction AND radiography) OR ALL (computed AND tomography) OR ALL (ct)))	395
Web of Science	(((“fractal dimension”[All Fields]) OR (“fractal analysis”[All Fields])) AND (((((“dental implants”[All Fields]) OR (“implant stability”[All Fields])) OR (“osseointegration”[All Fields])) OR (“bic”[All Fields])) OR (“bone to implant contact”[All Fields]))) AND ((((((((((“periapical radiograph”[All Fields]) OR (“iopa”[All Fields])) OR (“panoramic radiographs”[All Fields])) OR (“opg”[All Fields])) OR (“orthopantomograph”[All Fields])) OR (“cbct”[All Fields])) OR (“cone beam computed tomography”[All Fields])) OR (“subtraction radiography”[All Fields])) OR (“computed tomography”[All Fields])) OR (“ct”[All Fields]))	17
Total		426

**Table 2:** Demographic Characteristics of the Included Studies.

Author and Year, Country	Study Design	Randomization Method	Comparison	Total Patients Evaluated	Total Patients Included	Implant Placed	Analysed (Fractal Dimension)	Implant Placed	Analysed (Comparison)	Gender Distribution (M/F)	Age Range (Years)/Mean	Smokers	OHS
Lee et al., 2010 [22] South Korea	Prospective Clinical Study	NR	RFA	22	22	52	52	52	52	14M/8F	22–67	N/R	N/R
Kulczyk et al., 2018 [23] Poland	Prospective Clinical Study	NR	RFA	32	32	50	50	50	50	N/R	N/R	excluded	N/R
Verhoeven et al., 2009 [24] Netherlands	Prospective Clinical Study	NR	NR	8	8	16	16	NR	NR	1M/7F	50–78	N/R	N/R
Abdulhameed et al., 2018 [27] UAE	RCT	N/R	RFA	22	22	22	22	22	22	8M/14F	20–40	excluded	Poor oral hygiene excluded
Gonzalez-Martın et al., 2012 [28] Philadelphia, USA	Prospective Clinical Study	NR	NR	3	3	3	3	NR	NR	N/R	18–80	>10/day excluded	N/R
Mu et al., 2013 [21] South Korea	Retrospective Clinical Study	NR	NR	48	48	72	72	NR	NR	23M/25F	33–77	N/R	Poor oral hygiene excluded
Sansare et al., 2012 [20] India	Retrospective Clinical Study	NR	NR	50	33	50	50	NR	NR	20M/13F	25–30	N/R	N/R
Hayek et al., 2019 [26] Lebanon	Prospective Clinical Study	NR	RFA	50	50	50	50	50	50	50M	20–50	Excluded	Professional prophylaxis completed before surgery
Veltri et al., 2007 [25] Italy	Prospective Clinical Study	NR	RFA	9	9	55	48	55	48	2M/7F	46–68	N/R	N/R
Diana et al., 2018 [19] India	RCT	Sequence of appearance at the study institution	RFA	31	29	41	39	41	39	18M/13F	28.5	Excluded	Poor oral hygiene excluded
Önem et al., 2012 [15] Turkey	Prospective Clinical Study	NR	Lacunarity and Feret Diameter	42	42	42	42	42	42	N/R	27–43	N/R	N/R
Suer et al., 2016 [16] Turkey	Retrospective Clinical Study	NR	RFA	NR	NR	52	52	52	52	19M/11F	42.2	N/R	Excluded
Zeytinoğlu et al., 2014 [17] Turkey	Prospective Clinical Study	NR	NR	76	76	198	198	NR	NR	44M/32F	38.4	N/R	N/R
Soylu E. et al., 2021 [18] Turkey	Retrospective Clinical Study	NR	NR	39	39	66	66	NR	NR	20M/19F	52.2	N/R	N/R

Legend: RCT = Randomized Clinical Trial; NR = Not Relevant; N/R= Not Reported; RFA = Resonance Frequency Analysis; OHS = Oral Hygiene Status.

**Table 3:** Interventional characteristics of the included studies.

Author	Surgical Technique Used	Type of Implant Used	Implant Loading	Region of Implant Insertion	Implant Dimensions	Radiograph Taken	Time at Which the Radiograph Was Taken	ROI	Intensity of the Binary Image (Pixel)	Method of FD Used	Software Used to Calculate FD
Lee et al., 2010 [22]	Non-submerged technique	External Hex Implants	Delayed	Maxillary (Mx) Incisor, Mx. Premolar, Mx. Molar, Mandibular (Mn) incisor, Mn. Premolar, Mn. Molar	4 mm	OPG	0	Total implant site	128	Tile-counting method	Scion Image
Kulczyk et al., 2018 [23]	Open, full-thickness flap protocol	Titanium Dental Implants	Delayed	Mx. and Mn. jaw	N/R	IOPAR	0	bone adjacent to the neck (ROI 1), middle (ROI 2), and apical (ROI 3) part implant, respectively.	128	N/R	ImageJ program
Verhoeven et al., 2010 [24]	N/R	Titanium Dental Implants	Delayed (Bar Retained Over Denture)	2 at anterior mandible	N/R	Oblique lateral cephalometric	0, 1, 3 months	Total implant site	256	N/R	Carl Zeiss Vision KS 400 3.0 image analysis system
Abdulhameed et al., 2018 [27]	N/R	SPI dental implant	Delayed	Mx. edentulous premolar area	L—9.5 mm, D—4 mm	IOPAR	0, 3, 6 months	First macro thread around the mesial (ROI I) and distal (ROI II) aspects of each implant	128	Box-Counting Method	ImageJ program
Gonzalez-Martin et al., 2012 [28]	Flapless Osteotomy	Astra Tech implants	Immediate	Mx. Incisors, Patient 1—22, Patient 2—21, Patient 3—12	diameter—4.5 mm diameter of apical portion—3.5 mm, length—Patient 1 &2—13 mm, Patient 3—11 mm	CBCT	0, 6 months	Circular ROI 10.7 mm at Implant’s apical 1.15 mm	128	Box-Counting Method	ImageJ program
Mu et al., 2013 [21]	Two-stage surgical protocol.	Internal hex implants (Astra Tech)	Delayed	Anterior and posterior maxilla, Posterior Mandible	lengths—8–13 mm, Diameter—3.5–5 mm	IOPAR	0, 12 months	Width of 1.0 mm adjacent to the implant-bone interface at first macro thread around the mesial and distal aspects of each implant.	128	Box-Counting Method	ImageJ program
Sansare et al., 2012 [20]	Two-step surgical protocol.	N/R	Delayed	23 were in the maxilla and 27 were in the mandible.	N/R	OPG	0, 3 months	Distal aspect of the peri-implant area on the post-implant radiographs. One side of the square was oriented parallel to the long axis of the implant, without including any part of the implant. The other side was placed at the level of the apical end of the implant.	80	Box-Counting Method	ImageJ program
Hayek et al., 2020 [26]	N/R	N/R	Delayed	Mx. molar-left—3, right—4, Mx. premolar-left—12 right—6, Mn. molar-right—11, left—6 Mn. premolar left—2 right—6	length—10 mm and diameter—4 mm	IOPAR	0 (pre- and post-operative)	Area of the recipient site of the implant on the preoperative radiograph and adjacent to the inserted implant area on the postoperative radiograph.	128	Box-Counting method	ImageJ software
Veltri et al., 2007 [25]	N/R	Brane-mark System Nobel Biocare	Delayed	Upper jaw	N/R	IOPAR	36 months (present)	Two ROIs were selected mesial and distal to each implant, positioned in close contact with the implant threads	128	Box-Counting method	ImageJ software
Diana et al., 2018 [19]	Sequential Drilling.	Osstem implant	Delayed	one or more non-restorable single-rooted teeth with sufficient bone volume	Diameter—3.5–4 mm, Length—11.5–16 mm	IOPAR	0, 1, 3, 6, 12 months	mesial and distal aspects of the implant, at the implant shoulder level, at the mid-implant level, apical level.	128	Box-Counting method	ImageJ software
Önem et al., 2012 [15]	N/R	N/R	N/R	Two unloaded implants in the premolar/molar area on one side of the mandible and at least one premolar and/or molar tooth on the contralateral side	N/R	OPG	Over a period of 9.8 weeks	Three rectangular ROIs of the same size (mesial crest, distal crest, and apical area,	128	N/R	ImageJ software
Suer et al., 2016 [16]	Single Staged	Straumann	Delayed	Mn. Premolar and Molar area	Diameter—4.1 mm, length—10 mm	OPG	18 months	0.5 mm larger than the implant	128	Box-Counting method	ImageJ software
Zeytinoğlu et al., 2015 [17]	Two-stage surgical protocol	Frialit	Delayed	Mn. molar/premolar	N/R	OPG	0, 3, 6, 12 months	Mesial, distal, and apical areas were selected for each implant (close to the neck and apex)	128	Box-Counting method	ImageJ software
Soylu E. et al., 2021 [18]	N/R	ITI, Implance, DYNA, Dentium, Bilimplant	Delayed	Mn. premolar/molar	Diameter—3.7–4.1 mm, Length—8–11.5 mm	OPG	0, 1 week; 1 and 3 months	Mesial, distal, and apical sites of the implants	128	Box-Counting method	N/R

Legend: N/R = not reported; Mx = maxillary; Mn = mandibular; ROI = Region of Interest; OPG = orthopantomography; IOPAR = intraoral periapical radiograph; CBCT = cone beam computed tomography.

**Table 4:** The result outcomes of Risk of Bias of non-randomized studies of the intervention.

Author	Confounding Bias	Selection Bias	Bias Due to Adjustment for Departures from Intended Interventions	Information Bias	Bias Due to Missing Data	Bias Due to Outcome Measurement	Reporting Bias	Overall
Lee et al., 2010 [22]	Serious	Moderate	Low	Low	Serious	Low	Low	Serious
	Smokers and Poor oral hygiene subjects were not excluded, neither they control it later in the study.	No selection bias. However, no information about follow-up time.	No deviation from the intended intervention	No missing or misclassified information	Calculated FD values were not tabulated, only plots were given.	The knowledge of the intervention used has not influenced the outcome measurement.	No selective reporting done	
Kulczyk et al., 2018 [23]	Low	Low	Low	Moderate	Low	Low	Low	Moderate
	Patients with the factors affecting implant stability were excluded.	No selection bias	No deviation from the intended intervention	No information on implant dimensions used, as well as the method of FD calculation.	No missing data.	The knowledge of the intervention used has not influenced the outcome measurement.	No selective reporting done	
Verhoeven et al., 2010 [24]	Serious	Low	Low	Moderate	Serious	Moderate	Low	Serious
	No control methods were used for the non-selection of factors which can lead to confounding. Smokers, poor oral hygiene and patients with systemic diseases not excluded.	No selection bias	No deviation from intended intervention	No information about implant dimensions used, as well as the method of FD calculation.	Calculated FD values were not tabulated, only qualitative changes were summarised.	Outcome assessors were not blinded to the intervention used.	No selective reporting done	
Gonzalez-Martın et al., 2012 [28]	Low	Serious	Low	Low	low	Low	Low	Serious
	Proper inclusion exclusion criteria were set, and no post intervention confounders present.	Only 3 patients were selected for the study with the selected criteria that would favour the study.	No deviation from the intended intervention	No missing or misclassified information	No missing data.	The knowledge of intervention used has not influenced the outcome measurement.	No selective reporting done	
Mu et al., 2013 [21]	Low	Low	Low	Moderate	Low	Low	Low	Moderate
	Subjects with good oral hygiene, under functional prosthesis, non-smokers, and systemically healthy were included.	No selection bias	No deviation from intended intervention	No information about the trabecular changes.	No missing data.	The knowledge of intervention used has not influenced the outcome measurement.	No selective reporting done	
Sansare et al., 2012 [20]	Serious	Low	Low	Serious	low	Low	Low	Serious
	Confounding factors were not controlled.	No selection bias	No deviation from intended intervention	No information about the type of implant used as well as the dimensions were not mentioned.	No missing data.	The knowledge of intervention used has not influenced the outcome measurement.	No selective reporting done	
Hayek et al., 2020 [26]	Low	Low	Low	Serious	low	Low	Low	Serious
	Smokers and systemically ill were excluded. Oral prophylaxis completed before surgery. Subjects were not under medication that may affect bone metabolism.	No selection bias	No deviation from intended intervention	No information about the surgical protocol, the type of implant used as well as the dimensions were not mentioned.	No missing data.	The knowledge of intervention used has not influenced the outcome measurement.	No selective reporting done	
Veltri et al., 2007. [25]	Critical	Low	low	Serious	low	Low	Low	Critical
	No proper criteria or controls mentioned for the confounding factors.	No selection bias	No deviation from intended intervention	No information about the surgical technique used as well as the dimensions of implants were not mentioned.	No missing data.	The knowledge of intervention used has not influenced the outcome measurement.	No selective reporting done	
Önem et al., 2012 [15]	Serious	Moderate	low	Critical	Low	Low	Low	Critical
	Only subjects with systemic diseases and with medications that may affect bone metabolism were excluded.	No mention of number of males and females	No deviation from intended intervention	No information about the surgical technique, type of implant used as well as the dimensions were not mentioned.	No missing data.	The knowledge of intervention used has not influenced the outcome measurement.	No selective reporting done	
Suer et al., 2016 [16]	Moderate	low	Low	Low	low	Low	Low	Moderate
	Smokers included.	No selection bias	No deviation from intended intervention	No missing or misclassified information.	No missing data.	The knowledge of intervention used has not influenced the outcome measurement.	No selective reporting done	
Zeytinoğlu et al., 2015 [17]	Moderate	low	Low	Moderate	Low	Low	Low	Moderate
	Few confounding factors such as smoking and poor oral hygiene not excluded.	No selection bias	No deviation from intended intervention	No information on implant dimensions.	No missing data.	The knowledge of intervention used has not influenced the outcome measurement.	No selective reporting done	
Soylu et al., 2021 [18]	Moderate	Low	Low	Moderate	Low	Low	Low	Moderate
	Few confounding factors such as smoking and poor oral hygiene not excluded.	No selection bias	No deviation from intended intervention	No mention of the software used for FD calculation.	No missing data.	The knowledge of intervention used has not influenced the outcome measurement.	No selective reporting done	

## Data Availability

Not Applicable.
